# Delta-Alpha EEG pattern reflects the interoceptive focus effect on interpersonal motor synchronization

**DOI:** 10.3389/fnrgo.2022.1012810

**Published:** 2022-10-25

**Authors:** Laura Angioletti, Michela Balconi

**Affiliations:** ^1^International Research Center for Cognitive Applied Neuroscience (IrcCAN), Università Cattolica del Sacro Cuore, Milan, Italy; ^2^Research Unit in Affective and Social Neuroscience, Department of Psychology, Università Cattolica del Sacro Cuore, Milan, Italy

**Keywords:** interoceptive attentiveness, EEG, delta, alpha, motor synchronization

## Abstract

Little is known about how the modulation of the interoceptive focus impacts the neural correlates of high-level social processes, such as synchronization mechanisms. Therefore, the current study aims to explore the intraindividual electrophysiological (EEG) patterns induced by the interoceptive focus on breath when performing cognitive and motor tasks requiring interpersonal synchronization. A sample of 28 healthy caucasian adults was recruited and asked to perform two tasks requiring interpersonal synchronization during two distinct conditions: while focusing on the breath or without the focus on the breath. EEG frequency bands (delta, theta, alpha, and beta band) were recorded from the frontal, temporo-central, and parieto-occipital regions of interest. Significant results were observed for the delta and alpha bands. Notably, higher mean delta values and alpha desynchronization were observed in the temporo-central area during the focus on the breath condition when performing the motor compared to the cognitive synchronization task. Taken together these results could be interpreted considering the functional meaning of delta and alpha band in relation to motor synchronization. Indeed, motor delta oscillations shape the dynamics of motor behaviors and motor neural processes, while alpha band attenuation was previously observed during generation, observation, and imagery of movement and is considered to reflect cortical motor activity and action-perception coupling. Overall, the research shows that an EEG delta-alpha pattern emerges in the temporo-central areas at the intra-individual level, indicating the attention to visceral signals, particularly during interpersonal motor synchrony.

## Introduction

To date, little is known about how the focus on the body signal, known as interoception, impacts the interpersonal synchronization mechanisms. As a broad area of interest, “social interoception” focuses on how interoception influences many types of social phenomena, such as self-other distinction (Palmer and Tsakiris, 2018), social cognition (conceived in the core components of theory of mind, empathy, and imitation; Gao et al., 2019), loneliness and social connection (Arnold et al., 2019) and emotional experience (Burleson and Quigley, 2021). However, there are limited studies focusing on interoception and interpersonal synchronization, especially in the neuroscientific field.

Among the different interoceptive components, Interoceptive Attentiveness (IA) is defined as “focused attention to a specific interoceptive signal for a set time interval” (Schulz, 2016; Tsakiris and De Preester, 2018). Former works demonstrated that the sustained IA on breath sensations, typical of breath awareness and mindfulness practices, increase the neuronal activation of interoception networks and has beneficial effects on emotional (Arch and Craske, 2006) and cognitive functions (Weng et al., 2021). Nevertheless, there is currently few research to determine if IA affects the neurophysiological underpinnings of interpersonal synchronization mechanisms.

Interpersonal synchronization is a necessary and an essential social activity that encourages affinities and cooperation during routine joint social interactions and stands on the basis of individual and social dynamics. In its broadest sense, it includes a range of social communication behaviors (Charman, 2011), including turn-taking, mimicry, collaborative attention, and non-verbal social communication that involves synchronizing time and content (Delaherche et al., 2012).

Neuroscientific studies exploring synchronization frequently adopt behavioral synchrony tasks that involve movement or language (Balconi and Vanutelli, 2017). In particular, the introduction of the hyperscanning paradigm in the neuroscientific field (Montague et al., 2002; Balconi and Vanutelli, 2017) enabled a deeper understanding of the neurophysiological principles underlying interpersonal synchronization. Thanks to this paradigm, the neurophysiological brain correlates of synchronization during motor and linguistic imitation tasks were examined in earlier investigations.

Specifically, the electroencephalogram (EEG) is a relatively easy-to-use, affordable technology for the collection of brain electrical impulses with a millisecond-level temporal accuracy. It is easier for individuals to communicate in an ecological and naturalistic continuous stream since EEG equipment emits less auditory noise than magnetic resonance scanners do. Even though the limited spatial resolution of EEG makes it difficult to pinpoint the precise location of particular brain activity, it nonetheless shows regional scalp activation (Balconi and Lucchiari, 2005). Given its advantages, EEG was previously used in hyperscanning studies requiring face-to-face synchronization.

About motor synchronization, concurrently the performance of joint-tapping motor tasks, the synchronization between the prefrontal areas of two interacting individuals were observed (Funane et al., 2011; Cui et al., 2012; Holper et al., 2012; Cheng et al., 2015; Baker et al., 2016; Pan et al., 2017). In a more complex and ecological motor task requiring two participants to play a guitar duo, more phase-synchronized theta and delta oscillations in frontal and central electrode sites when playing a brief melody were observed: this result was interpreted as a coordinated firing of neuronal assemblies in the motor and somatosensory cortex, which regulate and coordinate motor activity, as well as frontal regions supporting social cognition (Lindenberger et al., 2009).

Also, during visually mediated social cooperation, oscillatory components in the alpha frequency range have been observed (Tognoli et al., 2007). Furthermore, modifications in the P3 Event-Related Potential component, which most likely represents low-frequency oscillations (e.g., in the delta range), have been found in the presence of interpersonally shared task representations (Sebanz et al., 2006). Limb and hand motions (Waldert et al., 2008), as well as sensorimotor integration (Caplan et al., 2003), also entail low-frequency oscillations. The frequency range up to 20 Hz appears to be engaged in sensorimotor interaction and interpersonal coordination, both of which are critical for interpersonal action coordination (Lindenberger et al., 2009). Recently, Müller et al. (2021) explored EEG interbrain synchronization during two neurofeedback tasks requiring interpersonal action coordination and showed a strong decrease of power spectra density in all frequency bands in the neurofeedback task conditions compared with the rest condition. However, the authors did not employ an interoceptive manipulation in their study.

About cognitive synchronization, by using the hyperscanning paradigm also live face-to-face interactive speech has been investigated (Jiang et al., 2012; Scholkmann et al., 2013; Liu et al., 2017; Hirsch et al., 2018; Zhang et al., 2018; Descorbeth et al., 2020). According to a recent comprehensive analysis of research using hyperscanning to study spoken language and communication (Kelsen et al., 2022), frontal and temporo-parietal areas supported the mirroring and mentalizing mechanisms that take place during communication activities.

During a human-human alternating speaking task, theta/alpha oscillation amplitudes were discovered to be amplified and synchronized between two participants in the same bilateral temporal and lateral parietal areas (Kawasaki et al., 2013). Additionally, compared to other dyads, those that had eye contact prior to class talks had the strongest alpha coherence (Dikker et al., 2017). According to Pérez et al. (2017), there was neuronal alignment with respect to alpha-band wave activity for listeners in the frontal area and speakers in the central region, as well as with respect to the theta band for listeners in the temporal region and speakers in the frontal region.

The EEG hyperscanning studies mentioned above did not foresee the evaluation of the manipulation of interoception, such as the request to focus on the breath during the execution of the task. To our best, there is still a shortage of works that examine the impact of IA on the EEG correlates of a single brain during a motor or cognitive synchronization task.

In two recent pilot studies, we have explored the effect of explicit IA manipulation on hemodynamic brain correlates during synchronization tasks by adopting a functional Near-Infrared Spectroscopy (fNIRS) (Angioletti and Balconi, 2022). According to the findings, setting the attention to the breath might produce a boosting effect on the brain's PFC response to synchronization, both for basic motor and cognitive synchronization tasks. Moreover, we have demonstrated that brain regions supporting sustained attention, such as the right PFC, were more involved when the intentional focus on the breath was required during the cognitive task requiring synchronization with a partner (Balconi and Angioletti, 2022).

More recently, in a second study, the effect of IA was tested by confronting a task involving interpersonal motor coordination framed with or without an explicit social goal (Angioletti and Balconi, 2022). In this work, results suggested that as the person consciously focuses on physiological interoceptive correlates and performs a motor task requiring synchronization with an explicit social framework there is a significant enhancement of neural activation of PFC areas that support shared intentionality, attentional focus, and high-order social processes.

Considering the direct connection between interoceptive correlates (and the focus centered on regulating them) and motor performance as well as the neuroanatomic proximity between the interoceptive and the motor areas, the results from the first study appeared unexpected. Additionally, these two pilot investigations recruited a small sample size, and the PFC was the only area of the brain that the fNIRS technology was applied on the PFC only, excluding other brain regions and the somatosensory cortical areas (Balconi and Molteni, 2016).

To our knowledge, there are no previous studies investigating the relationship between interoception, synchronization, and EEG frequency waves, so further studies are needed to disentangle the EEG correlates of IA effects on cognitive and motor tasks.

Therefore, the current study will examine the intraindividual EEG cortical patterns that characterize the interoceptive focus on the breath during cognitive and motor tasks requiring interpersonal synchronization. To achieve this aim, participants were required to concentrate on their breath while simultaneously performing a socially framed motor and cognitive synchronization task.

Given these premises and the boosting effect of the focused attention on the breath state over cognitive functioning (Angioletti and Balconi, 2022), we suppose that the EEG frequency bands related to the synchronization tasks could be significantly more present in the focus on the breath condition.

Moreover, we were interested in exploring if this interoceptive manipulation (i.e., the focus on the breath condition) has a significant impact on the motor or cognitive synchronization.

Specifically, we hypothesized to detect low-frequency oscillations (delta and theta) in frontal and central electrode locations during the performance of the motor synchronization task in the focused condition, which may signify coordinated and regulated motor activity (Lindenberger et al., 2009).

Regarding the cognitive synchronization task, a significantly higher presence of mostly alpha and theta band wave activity in the temporal and parietal areas during the task is expected, which is consistent with Kawasaki et al. (2013) study. In comparison to the control condition, this pattern should notably be reinforced in the focus on the breath condition.

## Methods

### Sample

The study sample was composed of 28 healthy participants [21 females and 7 men; age mean (M) = 24.2; Standard Deviation (SD) = 3.11] recruited by means of a non-probabilistic convenience sampling method. Given that the examined phenomenon is relatively novel in the field of social neuroscience and the literature did not provide systematic repeated evidence, it was not possible to exploit former references to estimate the size of the expected significant effects. Therefore, to estimate a minimum needed sample size, we ran a priori power analysis for repeated measures ANOVA and a total sample size (with alpha error probability = 0.05 and power 0.80) of 27 was the minimum for detection of a significant within effect or interaction between factors (G^*^Power 3.1 software, Heinrich-Heine, Germany; Faul et al., 2007). Exclusion criteria for participation in the study encompassed physiologic conditions such as chronic or acute pain, severe medical and chronic diseases, seizures, traumatic brain damage, pregnancy, prior meditation experience, and any mental or neurologic abnormalities. All of the people who took part in the experiment were right-handed and had a normal or corrected-to-normal vision. All participants signed a written informed consent form before the experiment and were told they would not be remunerated. The approval for this study was provided by the Department of Psychology at the Catholic University of the Sacred Heart of Milan, in accordance with the Declaration of Helsinki (1964).

### Experiment procedure

A researcher was in charge of giving the experimental instructions and executing the synchronization tasks, while the participants were seated in the same dimly lit room. EEG was adopted to capture a baseline resting period of 120 s prior to the start of the experimental procedure.

Participants imitated the experimenter in the performance of two synchronization tasks while their EEG activity was constantly recorded.

The first synchronization task consisted in a motor synchronization task in which participants had to synchronize their finger movements with the experimenter sitting in front of them for a total duration of 3 min. The participants were specifically asked to place their hands on the table in the prone position, with the fingers spaced about 1 cm apart and their elbows resting on the surface. They raise the fingers of their dominant hand and tap the table with their thumb, little, ring, middle, and index fingers. They were not told to carry out this movement at a certain tempo or to lift their fingers as high as they could. The only request was to synchronize their fingers movement with the movement made by the researcher seated in front of them. The average number of loops—understood as the number of times for an entire finger tapping sequence- was 60.

While the cognitive synchronization task consisted of a spelling task in which participants had to synchronize with the experimenter sitting in front of them. Four syllables, “LA” “BA,” “CA,” and “DA” were to be pronounced consecutively and alternatively by the subject; for example, the experimenter said “BA” and then the participant said “BA” and so on. The speech patterns were not predetermined in advance. Without breaks, each linguistic synchronization task session lasted 3 min. For the 3 min, the average number of loops—the number of times from “LA” to “DA”—was at least 45. For a detailed overview of these experimental tasks, see Balconi and Angioletti (2022).

Moreover, to stress the shared intentionality and increase ecological validity, both tasks were socially framed by specifying that they need to synchronize during these tasks to develop greater teamwork skills.

The two synchronization tasks were executed by the whole sample in two distinct conditions: the focus on the breath (explicit IA) condition and no focus on the breath, as the control condition (Balconi and Angioletti, 2021a,b). While performing the task during the focus on the breath condition, participants were instructed to pay attention to their breath as follows: “During this task, we ask you to concentrate on your breathing. Try to pay attention to how you feel and whether your breathing changes as you complete the activity.” There were no additional instructions to pay attention to the breath in the control (i.e., no focus on the breath) condition.

**Figure 1:**
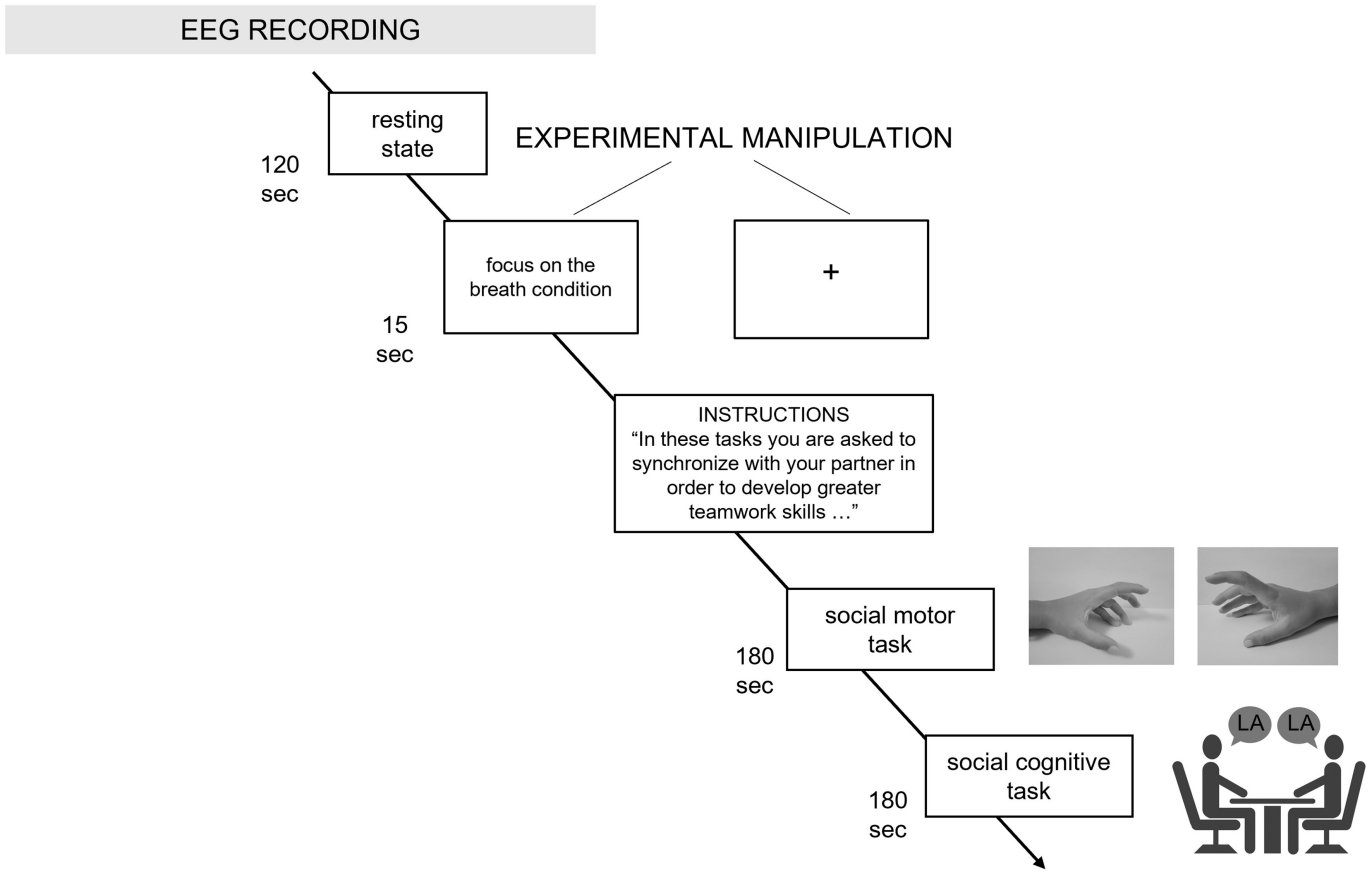
Experimental procedure. The procedure and the duration of each experimental step is graphically described in the figure, along with the baseline, experimental instructions, social motor, and social cognitive synchronization tasks.

To avoid biases brought on by sequence effects, the order in which the tasks were completed was randomized and counterbalanced. Less than 30 min were spent on the experiment as a whole (including the introduction to the experiment, the EEG montage, the baseline and tasks execution in both experimental conditions) (Figure 1).

### Electrophysiological data collection and biosignal analysis

**Figure 2:**
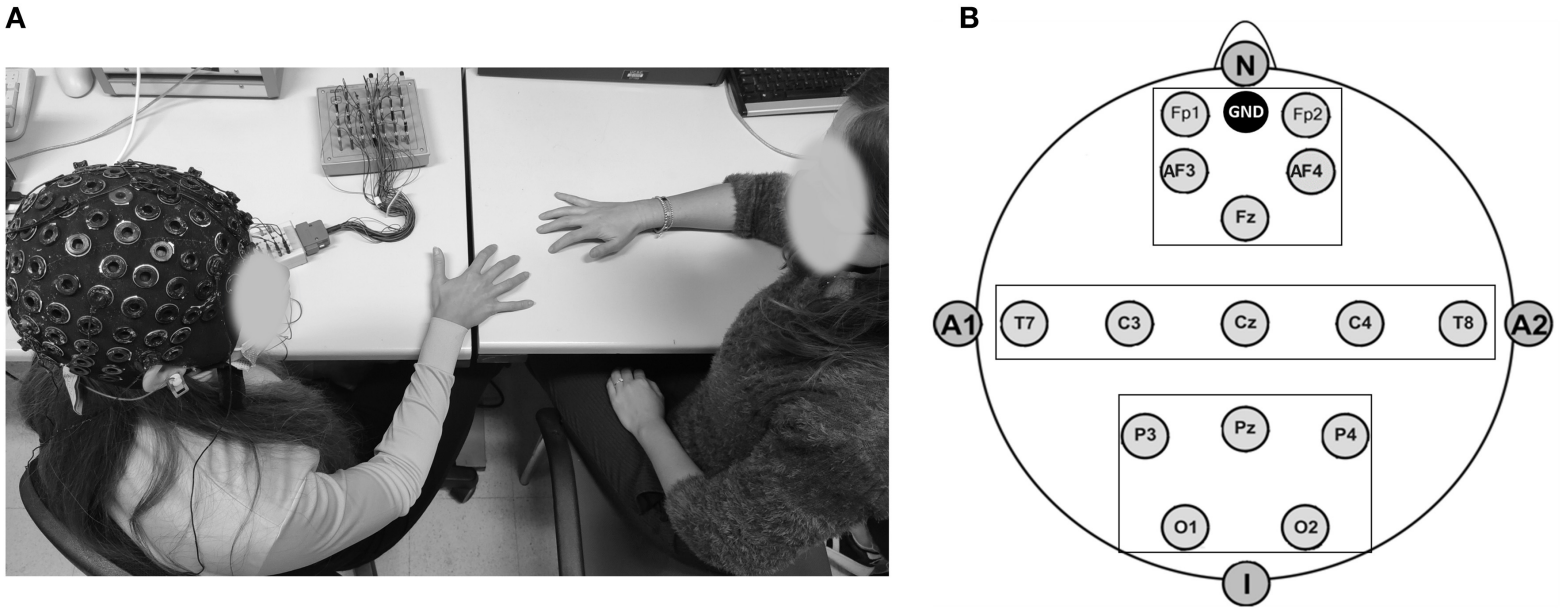
**(A,B)** EEG setting for data collection and layout adopted for the study. **(A)** Experimental setting displaying the participant wearing the EEG cap and performing the motor synchronization task with the experimenter. **(B)** Montage design included 15-electrodes positioning according to the 10–20 International System (Jasper, 1958) and divided in three main regions of interests: antero-frontal, temporo-central, and parieto-occipital.

To gather EEG data throughout task execution, a 32-channel amplifier (SYNAMPS system) and acquisition program (NEUROSCAN 4.2) were employed. EEG was recorded using an ElectroCap equipped with Ag/AgCl electrodes at active scalp locations referred to as the earlobes (10/20 International system of electrode placement) (Jasper, 1958; see Figure 2A). The following: Fp1, Fp2, AFF5h, Fz, AFF6h, T7, C3, Cz, C4, T8, P3, Pz, P4, and O1 and O2 were the 15 electrodes used in the EEG montage. Two EOG electrodes were further positioned on the outer canthi to track eye movements. Each subject's electrode impedance was assessed and kept under 5 kΩ prior to data collection. Data were sampled at 500 Hz and offline filtered with an IIR bandpass filter with a slope of 0.01–20 Hz (48 dB/octave). The signal was then separated and visually inspected for ocular, muscle, and movement artifacts. On segments free of artifacts, the Fast Fourier transform (Hamming window, resolution: 0.5 Hz) was used to calculate the average power spectra. After that, the average power for the main EEG frequency bands was computed (Delta 0.5–3.5 Hz, Theta 4–7.5 Hz, Alpha 8–12.5 Hz, and Beta 13–30 Hz). At the beginning of the experimental procedure, a 120 s baseline of resting was recorded.

Three areas of interest (Regions Of Interest, ROI) combining frontal (F: Fp1; Fp2; AF3; AF4), temporo-central (TC: T7; T8; C3; C4), and parieto-occipital (PO: P3; P4; O1; O2) electrodes were taken into consideration during the statistical analysis of the data (Balconi and Angioletti, 2021b; Figure 2B).

### Statistical analysis

Repeated measures ANOVA with independent within factors *Task* (2: motor and cognitive) × *Condition* (2: focus and no focus) × *ROI* (3: antero-frontal, temporo-central, and parieto-occipital) was applied to the dependent EEG frequency bands data, one for each frequency band (delta, theta, alpha, and beta). Pairwise comparisons were exploited to evaluate the significant interactions, the Bonferroni correction was used to reduce potential biases in repeated comparisons. All ANOVA tests employed Greenhouse-Geisser epsilon to adjust the degrees of freedom as necessary. The kurtosis and asymmetry indices were also used to evaluate the normality of the data distribution. The partial eta squared (η^2^) indices were used to assess the magnitude of statistically significant effects. Potential biases related to gender were checked for and excluded. No statistically significant main and interaction effect including gender were observed; then such variable was not included in below-reported analyses.

## Results

**Figure 3:**
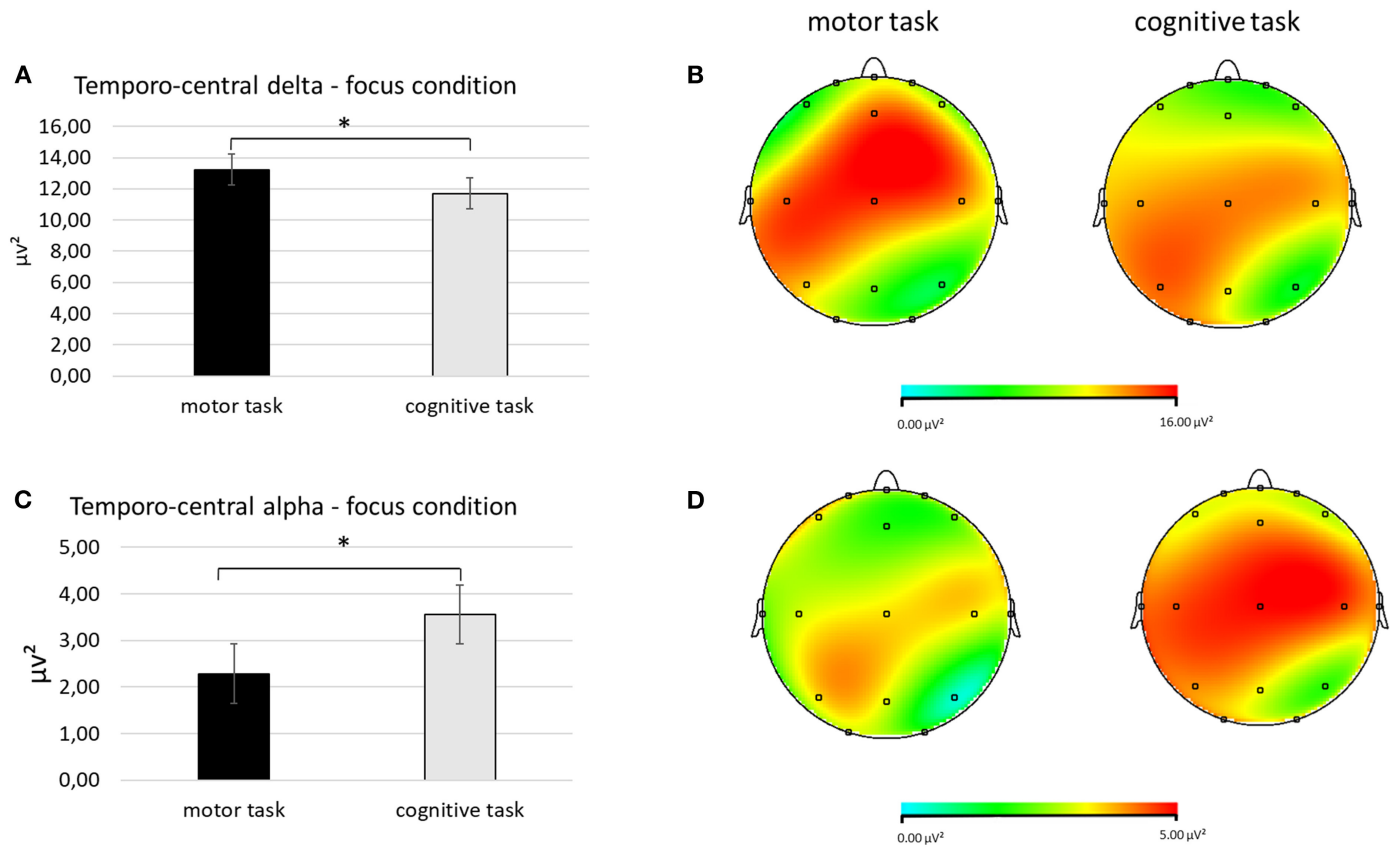
**(A–D)** Electrophysiological outcomes for delta and alpha bands. **(A)** The bar chart shows temporo-central delta mean values in the focus condition during the two synchronization tasks. **(B)** The red area represents greater delta power in the focus condition during the motor (left head) compared to the cognitive synchronization task (right head). **(C)** The bar graph displays the significant increase of temporocentral alpha values in the focus condition when performing the cognitive compared to motor synchronization task. **(D)** The red area represents greater alpha power in the focus condition during the cognitive (right head) compared to motor synchronization task (left head). For all charts, bars indicate ±1 Standard Error (SE); all asterisks mark statistically significant differences, with *p* ≤ 0.05.

First, regarding delta band, a significant interaction effect *Task* × *Condition* × *ROI* was found [*F*_(2, 27)_ = 8.67, *p* < 0.01, η^2^ = 0.278]. Pairwise comparisons revealed greater mean delta values in the temporo-central ROI during the focus condition in the motor compared to cognitive task [*F*_(1, 27)_ = 7.65, *p* < 0.01, η^2^ = 0.265; Figures 3A,B].

Secondly, for alpha band, it was detected a significant interaction effect *Task* × *Condition* × ROI [*F*_(2, 27)_ = 8.56, *p* < 0.01, η^2^ = 0.270]. Pairwise comparisons showed lower mean alpha values in the temporo-central ROI during the focus condition in the motor compared to the cognitive task [*F*_(1, 27)_ = 8.71, *p* < 0.01 η^2^ = 0.254; Figures 3C,D].

No significant effects were found for the theta and beta bands and no other significant effects were found.

## Discussion

This study aimed at examining the intraindividual EEG cortical patterns that characterize the interoceptive focus on the breath during cognitive and motor tasks requiring interpersonal synchronization. To achieve this aim, participants were required to concentrate on their breath while simultaneously performing a socially framed motor and cognitive synchronization task. The main results of the study were observed for the delta and alpha bands. Notably, higher mean delta values and alpha desynchronization were observed in the temporo-central area during the focus on the breath condition when performing the motor compared to the cognitive synchronization task.

These results partially confirmed our first hypothesis suggesting the presence of low-frequency oscillations (delta and theta band) in frontal and central electrode locations during the performance of the motor synchronization task in the focused condition, which may signify coordinated and regulated motor activity (Lindenberger et al., 2009).

In fact, it appears that motor synchronization occurs in specific areas (i.e., in the temporo-central area) and that this synchronization in amplified and mainly involves low frequency bands, which reflect a synchronous trend probably produced by the focus on breathing (as partially suggested by studies on meditation; Tei et al., 2009). Specifically, we observed an EEG pattern involving the increase of delta and decrease of alpha bands as an effect of the interoceptive focus during the motor synchronization task compared to the cognitive synchronization task. This effect suggests the impact of the interoceptive mental focus on motor synchronization correlates.

Also, our second hypothesis concerning the cognitive synchronization task, consistent with Kawasaki et al. (2013) study, and supposing a significantly higher presence of mostly alpha and theta band wave activity in the temporal and parietal areas during the focus on the breath condition was verified to some extent and discussed below.

Regarding the first result, for the delta band, an increase of the power values in the temporo-central regions during the focus on the breath condition and the motor compared to the cognitive synchronization task was observed and partially confirmed our first hypothesis.

Firstly, there is still a scarcity of research regarding the functional relevance of the delta band in this field. According to previous research on Zen and Qi-Gong (Tei et al., 2009), when people meditate, their EEG correlates show an increase in frontal delta power, which is likely a marker that cognitive engagement is being inhibited and that they are more able to detach from the experience. It is interesting to note that the rise in delta oscillation during mental activities was also correlated with functional cortical deafferentation, or suppression of the sensory afferences that interfere with internal focus (Harmony, 2013). Therefore, the attention on the breath instruction may be largely responsible for the manifestation of this frequency range.

Moreover, motor delta oscillations seem to shape the dynamics of motor behaviors and motor neural processes (Morillon et al., 2019). With respect to its expression in the temporo-central areas, an earlier research found delta oscillations in frontal and central electrode locations as an indicator of coordinated and monitored motor activity (Lindenberger et al., 2009). Therefore, it may be conceivable that the synergy experienced during the motor synchronization task is related to the temporo-central presence of delta in this particular circumstance. However, it is of relevance to note that this impact is only shown in the condition when the focus is on the breath (this difference is not seen in the no attention on the breath condition), indicating that this effect may be created (or made evident), particularly in the case of interoceptive concentration. These factors suggest that the interoceptive concentration may have encouraged the activation of this marker, which favors motor rather than cognitive synchronization (the latter here operationalized with a linguistic synchronization task).

Differently from what was hypothesized, significant results were detected only for delta and not for theta band, future studies are needed to disentangle the lack of results.

However, as a second and additional result to what we first hypothesized, a significant decrease of the alpha band in temporo-central regions during the focus in the breath condition and while performing the motor synchronization compared to the cognitive task was observed, leading to a two-fold interpretation based on the task executed.

Modulations of the alpha rhythm have been consistently reported to be implicated both in self-other entrainment tasks associated with interpersonal coordination (Tognoli et al., 2007; Dumas et al., 2010; Konvalinka et al., 2014; Novembre et al., 2016) as well as during action observation (Caetano et al., 2007; Arnstein et al., 2011), especially if the viewer has motor experience with the observed action (Cannon et al., 2014; Quandt and Marshall, 2014).

With reference to the motor task, before, research suggested that the suppression of the sensory-motor alpha rhythm, also known as event-related desynchronization (ERD), during action execution and observation, could be caused by a neurophysiological mechanism of motor resonance, the mirror mechanism (Streltsova et al., 2010). This mechanism is most likely due to the activation of neurons found in the premotor and posterior parietal cortices of macaque monkeys that have properties resembling mirror neurons (Di Pellegrino et al., 1992; Gallese et al., 1996; Rizzolatti et al., 1996; Balconi and Bortolotti, 2012). Also, alpha band desynchronization was observed during generation, observation, and imagery of movement and is considered to reflect cortical motor activity and action-perception coupling during kinematic regularities (Meirovitch et al., 2015). Suppression of oscillations within the alpha frequency range is an index of cortical excitability (Sauseng et al., 2009) and enhances the efficiency of cognitive processing (Klimesch, 2012).

On the other hand, the alpha power increase during the cognitive synchronization is partially in line with our second hypothesis and previous studies showing an amplification and synchronization in the same bilateral temporal and lateral parietal areas between two participants involved in an alternate speech (Kawasaki et al., 2013). Further, dyads that made eye contact before class discussions exhibited the highest alpha coherence in comparison to other pairs (Dikker et al., 2017). Pérez et al. (2017) found that speakers in the central region and listeners in the frontal area showed neuronal synchronization with regard to alpha-band wave activity.

Given this evidence, it might be plausible that the interoceptive focus on the breath might have led to a distinct effect based on the task executed. On the one hand, it may have enhanced alpha suppression during the motor synchronization task here interpreted as cortical excitability and enhanced efficiency of cognitive processing. This evidence constituted an additional result with respect to what we initially hypothesized for the motor synchronization task and this interpretation will have to be confirmed by future studies.

On the other hand, the focus on the breath condition might have promoted the alpha power presence over the temporo-central regions during the cognitive synchronization task, consistently with previous studies employing the joint task requiring verbal synchronization and engaging the temporal regions that support the mirroring and mentalizing mechanisms that take place during communication activities. This evidence, although not statistically significant, partially supports our second hypothesis. However, contrary to what was expected, we did not detect a significantly higher presence of theta band wave activity in the temporal and parietal areas during the cognitive synchronization task in the focus on the breath condition. Therefore, further works are needed to verify *if and what* are the effects of an interoceptive focus on the breath while performing a cognitive synchronization task.

Overall, this research shows that an EEG delta-alpha pattern emerges in the temporo-central areas at the intra-individual level, indicating the attention to visceral signals, particularly during interpersonal motor synchrony. Also, the increase of alpha band over temporo-central regions during the focus on the breath condition while executing the cognitive synchronization task is in line with previous studies.

Despite this study could be consdered one of the first work exploring the interoceptive focus on the breath, interpersonal synchronization, and EEG frequency bands as markers of these processes, some limitations should be taken into consideration. First, the sample size could be increased to generalize the present results. To control participants' interoceptive skills, further exclusion criteria in the sample selection process could be included: such as the use of self-report measures (e.g., the Body Perception Questionnaire; Cabrera et al., 2018) or behavioral methods [e.g., a respiratory sensitivity task (Garfinkel et al., 2016) or the Heartbeat counting task (Desmedt et al., 2018)]. Moreover, the use of an EEG hyperscanning paradigm to collect the neurophysiological data from the other member of the dyad, might be useful to grasp the interactional dynamic and be beneficial for future research. Given the limited EEG spatial localization, spatial source localization could be improved by introducing an EEG-fNIRS co-registration.

To conclude, this study suggested that the interoceptive focus on the breath expands the effects of interpersonal synchronization and may have a predominant impact on motor synchronization tasks, helping identify patterns and brain sources that are actually trained and can be empowered by breath awareness practices during joint synchronization tasks. This evidence might be of interest to professionals proposing breath awareness practices combined with motor or cognitive synchronization exercises, such as physiotherapy and logotherapy rehabilitation exercises, but also synchronized sports performance.

## Data availability statement

The raw data supporting the conclusions of this article will be made available by the authors, without undue reservation.

## Ethics statement

The studies involving human participants were reviewed and approved by Department of Psychology, Catholic University of the Sacred Heart, Milan, Italy. The patients/participants provided their written informed consent to participate in this study.

## Author contributions

LA and MB contributed to the conception, design of the study, manuscript revision, read, and approved the submitted version. LA wrote the first draft. All authors contributed to the article and approved the submitted version.

## Conflict of interest

The authors declare that the research was conducted in the absence of any commercial or financial relationships that could be construed as a potential conflict of interest.

## Publisher's note

All claims expressed in this article are solely those of the authors and do not necessarily represent those of their affiliated organizations, or those of the publisher, the editors and the reviewers. Any product that may be evaluated in this article, or claim that may be made by its manufacturer, is not guaranteed or endorsed by the publisher.
